# Psychopathology in Adolescent Alcohol Abuse and Dependence

**Published:** 1998

**Authors:** Duncan B. Clark, Oscar G. Bukstein

**Affiliations:** Duncan B. Clark, M.D., Ph.D., is an associate professor of psychiatry at the University of Pittsburgh School of Medicine and scientific director of the Pittsburgh Adolescent Alcohol Research Center (PAARC), and Oscar G. Bukstein, M.D., M.P.H., is an associate professor of psychiatry at the University of Pittsburgh School of Medicine and clinical director of PAARC, Pittsburgh, Pennsylvania

**Keywords:** antisocial personality disorder, emotional and psychiatric depression, AODD (alcohol and other drug use disorder), affective psychosis, comorbidity, dual diagnosis, adolescent, psychiatric care, addiction care, patient assessment, treatment method, literature review

## Abstract

Adolescents who abuse or are dependent on alcohol often have coexisting mental disorders. These disorders may both precipitate alcohol use disorders and result from them. In addition, both types of disorders may arise independently in adolescents at high risk. Mental disorders that commonly co-occur with alcohol use disorders in adolescents include antisocial disorders, mood disorders, and anxiety disorders. Treatment programs for adolescents with alcohol use disorders should seek not only to eliminate alcohol and other drug use but also to improve the symptoms of other mental disorders.

Adolescents with alcohol use disorders (AUDs) (e.g., alcohol abuse or dependence^1^) have high rates of coexisting (i.e., comorbid) psychopathology (i.e., mental disorders other than alcohol and other drug use disorders). Common comorbid psychopathologies include those that interfere with social functioning (e.g., antisocial disorders) and disorders that cause severe depression or increase anxiety (i.e., negative-affect disorders) (Bukstein et al. 1989; Clark and Neighbors 1996). One study found that more than 80 percent of adolescents who were dependent on or abused alcohol also had some other form of psychopathology (Rohde et al. 1996). Among a group of alcohol-dependent adolescents participating in treatment, 89 percent also had conduct disorder (i.e., an antisocial disorder characterized by aggression, destruction of property, deceitfulness or theft, and the violation of rules), major depressive disorder (i.e., a negative-affect disorder characterized by severe bouts of depression), or both (Clark et al. 1997). Understanding the effects of comorbid psychopathology on the development and course of AUDs may enhance preventive and treatment interventions for adolescents with AUDs.

The development of an AUD in adolescence may be an important indicator of other problems. Clark and colleagues (1998*c*) found that compared with men who developed substance use disorders (SUDs) (i.e., alcohol and other drug use disorders) as adults, adolescent males with SUDs and male adults who developed SUDs as adolescents had higher rates of disruptive behavior disorders and major depression as well as more rapid progression from first use to substance dependence.

Theories that attempt to explain the development of AUDs in adolescents have typically proposed that the presence of psychopathology increases the adolescent’s risk of developing an AUD by either precipitating the onset of an AUD in vulnerable people or exacerbating mild alcohol problems (Zucker 1987). Conversely, AUDs may influence the development of psychopathology through similar mechanisms (Martin and Bates 1998). Psychopathology and AUDs also may be indirectly linked by shared risk factors (i.e., they may coexist in a person because the person is at risk for both, not because one influences the other) (see figure).

This article reviews two types of mental disorders common in adolescents with AUDs: antisocial disorders, such as conduct disorder, and negative-affect disorders, such as major depressive disorder. (For a discussion of attention-deficit hyperactivity disorder [ADHD], see article by Wilens, pp. 127–130.) For each disorder, this article provides definitions, discusses the observed relationships to AUDs, and offers implications for treating adolescents with the disorder.

## Antisocial Disorders

### Definitions

Antisocial disorders include conduct disorder, oppositional defiant disorder (ODD), and antisocial personality disorder. The American Psychiatric Association’s *Diagnostic and Statistical Manual of Mental Disorders, Fourth Edition* (DSM–IV) (APA 1994) defines conduct disorder, the most common form of psychopathology seen in adolescents with AUDs, as a pattern of behaviors that violate the basic rights of others or major age-appropriate social rules. Behaviors that may indicate the presence of conduct disorder are classified into four categories: (1) aggression to people and animals, (2) destruction of property, (3) deceitfulness or theft, and (4) serious violations of rules. Severe conduct disorder is often preceded by the development of ODD (Loeber et al. 1993). In DSM–IV, the diagnostic criteria for ODD encompass less severe antisocial behavior than conduct disorder and include arguing, losing one’s temper, defying rules, deliberately annoying others, blaming others for one’s behavior, and inappropriate anger or vindictiveness (APA 1994). The DSM–IV diagnostic criteria for antisocial personality disorder, common in adults with AUDs, require a history of conduct disorder with onset by age 15 as well as multiple displays of antisocial characteristics in adulthood (APA 1994).

### Relationship of Antisocial Disorders to AUDs

Conduct disorder often predates and predicts alcohol use or an AUD (Clark et al. 1998*b*; Lynskey and Fergusson 1995) and may, in fact, contribute to the development of a problem with alcohol (see figure). Conduct disorder also may be a factor in the relationship between ADHD and AUDs (see article by Wilens, pp. 127–130). Adolescents with conduct disorder may be more likely to “act out” (i.e., to have poor behavioral inhibition). They also frequently seek new experiences (i.e., have increased novelty seeking). Consequently, they may begin drinking at an early age and therefore have increased risk for developing a problem with alcohol (Lewis and Bucholz 1991).

Conversely, an AUD also may facilitate antisocial behavior and precipitate ODD and conduct disorder (see figure). For example, alcohol use may contribute to poor judgment and association with delinquent peers, both of which can increase antisocial behaviors.

A third theory of the relationship between conduct disorder and AUDs suggests that each disorder shares common risk factors (see figure). According to this explanation, known as problem behavior theory, the development of both antisocial behaviors and early alcohol involvement can be accounted for by a combination of environmental characteristics—including family, socioeconomic, and parental factors—and individual characteristics that increase the adolescent’s vulnerability to these problems. Alcohol use is thus conceptualized as one of a number of deviant behaviors resulting from common risk factors, such as poor parental support and supervision, deviant peer group association, and low academic achievement. The shared risk factors may act either independently or synergistically to affect the severity and outcome of an AUD and the concurrent deviant social behavior. Several long-term studies of adolescents and young adults have supported problem behavior theory (Donovan and Jessor 1985).

### Treatment

Among adolescents with AUDs, those with conduct disorder are more difficult to treat than those without conduct disorder. The presence of conduct disorder predicts greater posttreatment alcohol consumption and the possibility of later development of antisocial personality disorder (Brown et al. 1996; Myers et al. 1995). Treatment strategies focusing on behavior change have met with some success for adolescents with co-occuring AUDs and conduct disorder. Those strategies include family interventions, contingency management programs (which offer incentives, such as retail items or special privileges, along with social reinforcement to encourage proper behavior), and social skills training (Bukstein 1995).

More intensive treatment strategies often are needed, however, for adolescents with serious conduct and alcohol-related problems. In such cases, multisystemic treatment (MST), which was developed by Henggeler and colleagues (1998) has proven effective. MST is an intensive multidimensional approach that combines family, peer, school, and community interventions with individual treatment to target multiple risk factors and problems. Treatment sessions are provided in the home and at times that are convenient to the family, resulting in fewer missed appointments and greater family involvement in treatment (Henggeler et al. 1996). Family interventions are designed to foster effective parenting and family cohesion using strategies integrated from multiple theoretical bases. Parents are directed to increase monitoring of their child’s relationships with peers and to promote improved school performance. Individual interventions with the adolescent target skill training and behavior change (Santos et al. 1995). MST has been evaluated in controlled trials and reported to be effective in reducing antisocial behaviors, substance-related arrests, and substance use (Henggeler et al. 1998).

**Figure:**
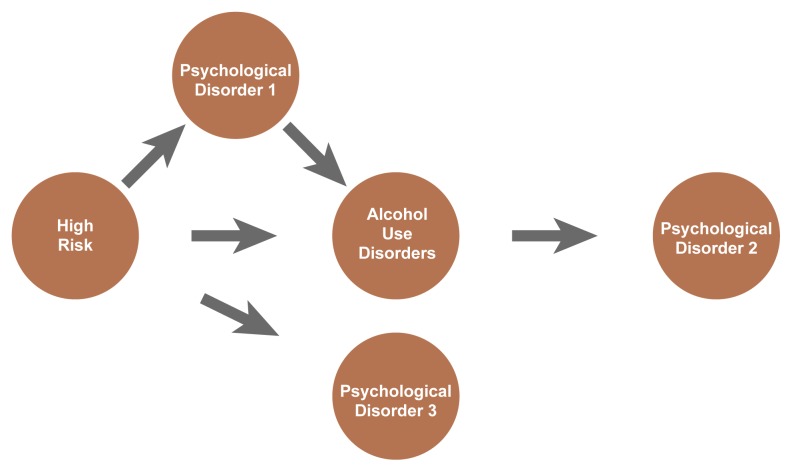
Pathways for risk, psychopathology, and alcohol use disorders (AUDs). “High risk” represents the various risk factors that have been linked to the development of AUDs and other mental disorders. Psychopathology (psychological disorder 1) may influence the relationship between high risk and an AUD. Alternatively, a comorbid mental disorder (psychological disorder 2) may result from an adolescent AUD. Risk factors also may increase vulnerability to psychopathology (psychological disorder 3) and AUDs independently, such that the prevalence of both psychopathology and AUDs would be increased in high-risk adolescents but might not be causally related.

In addition to treatment approaches focusing on behavior change, treatment for adolescents with conduct disorder and AUDs also may include the use of medications. Adolescents with conduct disorder often exhibit impulsivity, aggression, or anxiety, all of which may be alleviated by medications. Indeed, the frequent comorbidity of conduct disorder with ADHD may suggest that those adolescents could benefit from medications that are effective for ADHD. Stimulants such as methylphenidate (Ritalin^®^) or dextroamphetamine can be effective (Klein et al. 1997), but their use is controversial because of the risk that such drugs may be abused or sold illegally. The use of pharmacological treatment for adolescents with conduct disorder has not been extensively studied (see sidebar by Solhkhah and Wilens, pp. 122–125). Research is needed to determine which adolescents will benefit the most from pharmacological treatment.

## Negative-Affect Disorders

### Definitions

Negative-affect disorders include disorders that cause mood disturbances and depression and those that increase anxiety. Major depression and post-traumatic stress disorder (PTSD) are two negative-affect disorders found to commonly occur with AUDs among adolescents. For adolescents, the DSM–IV diagnostic criteria for major depression include depressed mood, irritable mood, or loss of interest in daily activities as well as at least five additional symptoms, such as insomnia, fatigue, guilt feelings, difficulty concentrating, and recurrent thoughts of suicide (APA 1994). Dysthymia, a less severe depressive disorder, may be diagnosed in adolescents who fall short of a diagnosis of major depression but who report a depressed or irritable mood for at least 1 year and the presence of two or more symptoms, such as suicidal thoughts or behavior, sleep or appetite problems, and low energy (APA 1994).

The DSM–IV defines PTSD as the development of specific symptoms following exposure to a traumatic event (i.e., experiencing or witnessing actual or threatened death or serious injury) to which the person responded with intense fear, helplessness, or horror (APA 1994). The characteristic symptoms that follow include the feeling of reliving the event through recurrent and intrusive recollections or recurrent distressing dreams; experiencing intense distress when exposed to cues that recall an aspect of the event; persistent avoidance of thoughts, feelings, conversations, activities, places, or people associated with the event; diminished interest in activities; and increased arousal, indicated by sleep difficulties, irritability, difficulty concentrating, or a heightened response to surprise. Anxiety disorders other than PTSD have not been clearly demonstrated to be associated with AUDs among adolescents (Clark and Neighbors 1996).

### Relationship of Negative-Affect Disorders to AUDs

The high rates of major depression and PTSD among adolescents with alcohol problems suggest that a high priority should be placed on understanding the relationships between those disorders and AUDs (Clark and Miller 1998). Major depression and PTSD are especially prevalent among female adolescents with AUDs (Clark et al. 1997; Bukstein et al. 1992). Histories of childhood physical abuse and sexual abuse are common among adolescents with negative-affect disorders and AUDs, and among adolescents with SUDs, those with major depression and PTSD have been found to have histories of childhood abuse (Deykin et al. 1992; Deykin and Buka 1997; Clark et al. 1998*a*). Physical and sexual abuse may influence the development of a negative-affect disorder, which in turn may lead to a SUD through efforts to self-medicate (Clark and Miller 1998). In this hypothesis, childhood abuse is the identified risk factor, and the negative-affect disorder precipitates the AUD. An alternative possibility is that childhood abuse contributes to both negative-affect disorders and AUDs and that neither disorder influences the development of the other. Additional research is needed to examine these potential explanations.

As with conduct disorder, AUDs may both contribute to and result from negative-affect disorders. Alcohol consumption can lead to major depression and anxiety disorders. In fact, abstinence often alleviates depression and anxiety symptoms in alcohol-dependent people (Brown et al. 1991; Brown and Schuckit 1988). In addition, AUDs may exacerbate PTSD symptoms by increasing the risk of being involved in a traumatic event, especially when intoxicated, and by intensifying PTSD symptoms through alcohol withdrawal (Stewart 1996).

### Treatment

Treatment for an adolescent with both a negative-affect disorder and an AUD should be preceded by a period of abstinence to determine whether treatment is needed for underlying mood and anxiety symptoms or whether those symptoms will improve with abstinence. When underlying or persistent negative-affect disorders are present in adolescents with AUDs, treatment should be directed at those disorders. Approaches may include psychological therapy or the use of medication. Psychological treatments for major depression and PTSD, such as approaches aimed at modifying the adolescent’s attitudes and behavior (i.e., cognitive behavior therapy) have been adapted for adolescents and may apply to those with comorbid negative-affect disorders and AUDs. Psychological treatment approaches are appropriate first-line interventions for those adolescents.

When psychological approaches are not successful, medications may be prescribed to help manage the symptoms of negative-affect disorders. The use of antidepressant medications in adolescents with AUDs is controversial. Fluoxetine (Prozac^®^) has been shown to be effective for depression in adolescents (Emslie et al. 1997) and for comorbid alcohol dependence and depression in adults (Cornelius et al. 1997). Those results suggest that fluoxetine and similar antidepressants also may be useful for adolescents with comorbid major depression and AUDs. As in other areas of comorbidity, further research is needed to determine when the use of medications is appropriate for adolescents with AUDs and negative-affect disorders and how those medications should be used.

## Considering Comorbidity in Assessment and Treatment

### Assessment

Assessment, an essential preliminary step toward treatment, is the evaluation of people who may have problems with alcohol or other drugs to determine whether a problem exists and, if so, how serious the problem is and what kind of treatment is appropriate. An assessment is often ordered for adolescents who exhibit problems with school, work, family, or peers. During assessment, it is important to consider the potential influences of both AUDs and other mental disorders. Clinicians evaluating and treating adolescents must be familiar with the typical signs and symptoms of adolescent AUDs, other SUDs, other mental disorders, and associated problems. The use of standardized assessment instruments or questionnaires is recommended, because informal assessment techniques can lead to incomplete and inaccurate diagnoses (Clark et al. 1995). Comprehensive assessment instruments designed for use in clinical settings are available to evaluate AUDs, other drug use disorders, mental disorders, and abuse history (e.g., Kiddie Schedule for Affective Disorders and Schizophrenia [K–SADS]).^2^ Difficult family relationships, the lack of adequate parental supervision, and deviant peer affiliations also are important influences to be considered in treatment planning (Clark et al. 1998*d*; Curran et al. 1997; Reifman et al. 1998). Comprehensive assessments take a considerable amount of time, but they provide valuable insights for planning and carrying out treatment.

### Treatment

The results of assessment are used in determining the type of AUD treatment that should be undertaken. Clinicians treating adolescents with AUDs should also be familiar with the treatment approaches available for treating mental disorders common in this population. A number of treatment strategies, including family interventions and cognitive behavior therapy, have been developed to address adolescent AUDs and common comorbid mental disorders (Bukstein 1995). Treating multiple disorders may require a combination of different types of therapies, such as MST (Henggeler et al. 1998). For example, an adolescent may need group-based therapy to address difficulties with problem-solving, anger control, and relapse prevention. In addition, the family may benefit from interventions designed to address issues of communication, parental control and supervision, and if relevant, the parents’ individual problems. Medications may be appropriate for managing problems such as ADHD and major depression. In adolescents with AUDs complicated by other disorders, interventions of greater intensity and duration are probably needed. Whether coexisting problems are addressed concurrently or consecutively, treatment for adolescents with both AUDs and comorbid mental disorders requires more time and resources than treatment for adolescents with a single disorder. Comprehensive approaches, such as MST (Henggeler et al. 1998), may hold the most promise for adolescents with comorbidity.

## Conclusion

Adolescent alcohol use may or may not lead to alcohol abuse or dependence, depending on the influence of multiple and complex factors (Tarter and Vanyukov 1994). Mental disorders may both precipitate and result from AUDs. In addition, both types of disorders may arise independently in adolescents at high risk. The multi-faceted relationships among comorbid mental disorders and AUDs in adolescents are not yet fully understood. Individual psychological characteristics interact with and are influenced by developmental stage, gender, family characteristics, and preventive as well as treatment interventions. Treatment programs designed for adolescents with AUDs should seek not only to eliminate alcohol and other drug use but also to improve the symptoms of other mental disorders by addressing family relationship difficulties and reversing the adverse effects of alcohol use on psychosocial functioning.

## Footnotes

1 Alcohol abuse and alcohol dependence are conditions defined by specific criteria in the American Psychiatric Association’s *Diagnostic and Statistical Manual of Mental Disorders, Fourth Edition*.

2 Kiddie Schedule for Affective Disorders and Schizophrenia (K–SADS) is available at www.wpic.pitt.edu/ksads.
